# New York Academy of Medicine

**Published:** 1880-12

**Authors:** 


### NEW YORK ACADEMY OF MUSIC.

*A New Female Urethral Dilator.*—The chairman exhibited a female urethral dilator which could also be used as a speculum. An objection that might be made to the instrument, namely, that the walls of the urethra might fall between its blades, could be overcome easily by introducing a probe and pressing the mucous membrane back. As we noticed, the instrument dilates more at its distal than its proximal extremity, a fact that favored dilatation of the urethra without doing injury to the meatus.

Dr. Caro reported two cases in which the instrument had been used with satisfactory result. A maiden lady, aged forty years, was taken with pain in the left hypogastric region. It recurred with each menstrual period and existed only during that time. Finally, she began to have painful micturition. Her urethra was dilated, and four small calculi removed from the bladder. The dilatation was not painful, and the recovery was complete. The second case was that of a young woman who began to suffer from painful micturition soon after marriage. The urethral and vaginal irritation became so great that sexual intercourse was impossible. There seemed to be a spasmodic irritation of the urethra; there was no evidence of inflammatory action, and a single dilatation relieved the patient at once of all discomfort. The dilatation was carried to the extent of about half an inch, when the instrument was allowed to remain in position, and it was soon expelled spontaneously.

Dr. Isaac E. Taylor remarked that the dilator presented by Dr. Caro was similar to an instrument which the late Dr. George T. Elliot and himself employed for examining the os uteri.

Dr. A. C. Post stated that he was the first to treat vaginismus by stretching the vagina. He also referred to one case of stricture of the female urethra which he treated successfully by dilatation.

Dr. Livingston referred to a case in which a lever-pessary had so compressed the urethra as to produce a partial but permanent obstruction of the canal.

*Inversion of the Uterus occurring in a Virgin*—Dr. I. E. Taylor reported a case of inversion of the uterus occurring in a patient aged forty years, whom he saw with Dr. Polk. There was not any evidence whatever that the woman had ever been impregnated. The uterus was about the size of a large orange. The inversion was complete. Dr. Taylor believed that the inversion had been gradual in its development, and that it began in the cervix.

*Abortion in the Tenth Week of Pregnancy without Hemorrhage.*—Dr. Taylor also reported a case in which a patient thirty-five years of age, had unusually severe pains during an abortion, and almost no blood was lost after the delivery of either the embryo or the placenta. After the embryo had been expelled, examination revealed a closed cervix. Laudanum was administered to modify the pains, which were exceedingly severe, and the patient was allowed to remain undisturbed. The placenta came away on the second day without hemorrhage, and there was no bloody discharge subsequently.

*Pregnancy Undisturbed by Manipulation and the Introduction of Instruments into the Uterus.*—Dr. Taylor also referred to a case in which the patient had one menstrual period after marriage, and subsequent to that had a slightly colored discharge, occasionally what seemed to be membranous remains passed off, and after a few days she flowed very freely. During the treatment to which she had been subjected, the case having been regarded as one of endometritis, the utering sound had been introduced, sponge-tents had been used, and various applications had been made to the cervix, yet abortion did not occur, although it was afterwards established that pregnancy must have existed at the same time.

*Cases Illustrating the Effect Produced by Chloroform in Labor.*—Dr. Taylor also narrated a case in which labor began at 5 A.M., and about 5.30 the os was found to be about the size of an old-fashioned sixpence. The pains were severe and continued so throughout nearly the entire day without increasing the dilatation to more than would be represented by a two-shilling piece, and finally they were quite irritating. He put the patient under the influence of chloroform, and within an hour she was delivered of a large, healthy child, weighing eleven pounds. The case illustrated the stimulant effects of chloroform. In another case the woman had been thirty-six hours in labor, and the head was upon the perineum. Chloroform had been given for several hours, and also ergot, of which Dr. Taylor was not made aware. He directed that the chloroform be withheld, and the child was rapidly delivered. There was no post-partum hemorrhage. The uterus was as round as a ball, and hard. The ergotic contraction had followed the partial removal of the anæsthetic.

Dr. Livingston remarked that he never gave chloroform in labor to the production of complete anæsthesia. He also referred to a case in which uterine pains ceased entirely whenever he entered the patient's room; and the phenomenon was repeated three or four times. He placed the woman partially under the influence of chloroform, and the labor progressed without further abnormal delay.

Dr. Livingston also referred to a case in which he was able to utilize a certain fact to his obstetric advantage. He was called in consultation to see a woman who had been in labor for some time, and in efforts at affecting delivery the head had been left in the cavity of the uterus, it having been separated from the body of the child by traction. The woman's pelvis was deformed. After making several unsuccessful attempts to catch the head, he noticed that, when the chloroform was partially withheld, the head occupied a comparatively fixed position, and he utilized that fact, withheld the anæsthetic, the head was grasped and held by the uterus sufficiently firm so that he was able to introduce the trephine, subsequently the cephalotrite, and complete the labor. It was the second case he had seen in which the head of the child had been separated from the body and left within the uterus.

Dr. Taylor referred to Dr. Goodell's statement, that a traction force of 200 pounds was required to pull the child's head off; and that he (Dr. G.) had never seen an instance in which the head was left within the uterine cavity. Dr. Taylor had seen three such cases, and he thought that if 100 pounds of traction force were applied there was very great danger of pulling the head of the child off.

*Spontaneous Version.*—Dr. Taylor also referred to a case, similar to several he had encountered, in which spontaneous version occurred after the waters had been evacuated four or five hours, and an attempt to deliver with the forceps had failed.

*Abdominal Tumor—Tapping—Removal of Purulent Fluid—Temporary Relief.*—Dr. A. C. Post narrated a case as follows: A woman, aged sixty years, came to the Presbyterian Hospital, from West Virginia, with a large abdominal tumor, and expected to have ovariotomy performed. After careful examination the conclusion was reached that it was a compound tumor of the uterus, composed of solid and fluid substance, and tapping was advised. The tumor was aspirated, and seven pounds of fluid, largely purulent, were removed. There had been no distinct inflammatory symptoms before, and there were none afterward. A compress and bandage were placed about the abdomen, and no unpleasant symptoms followed. The patient was very much relieved, and a few weeks subsequently, returned to her home, feeling quite comfortable.

*Case for Diagnosis.*—Dr. Livingston related a case with the following history: An apparently healthy, robust girl, aged fourteen years, suddenly felt that her clothes were wet over the left hip and buttock. On examination, a liquid, like serum, was seen oozing from one spot, and about two quarts flowed out. The fluid appeared only during the day, and at night the flow abated. There was not the slightest change in the appearance of the skin as compared with that on any other part of the body, and the discharge gradually subsided. At the end of seven months the same thing was repeated. In the month of August (1880) it occurred for the third time, and about three weeks ago it appeared for the fourth time. Dr. Livingston then examined the patient very carefully, but, for the time the discharge had ceased. He did not find anything abnormal. The next morning the discharge reappeared, and he then found, by the aid of a glass, a slightly enlarged follicle with an apparent opening, but it was not large enough to admit the end of the finest lachrymal probe. After using considerable force, the point of the instrument was made to enter slightly, fluid began to flow more freely, and had very much the appearance of synovial fluid, but it was not at all tenacious, nor were the clothes at all stiffened by being saturated with it, and afterward dried. The fluid could be seen coming out drop by drop, and so it continued to do so until by evening eight or ten napkins had been wet thoroughly. He estimated the quantity of fluid discharged at two or three pints. He enlarged the opening so as to permit the introduction of the nozzle of a small syringe, and injected warm water, but not more than a teaspoonful could be made to enter, which gave the girl pain extending down her thigh. Otherwise she was apparently in perfect health. When ten years of age she slid down hill considerably one day, squatting and sliding upon her feet. The next morning she was taken with very severe pain in the left hip, but she recovered, after a short confinement in bed, and with the use of the limb perfect. She subsequently attended dancing school, and it was not known that her limb was in any way unsound when the phenomenon referred to appeared.

